# Cryptococcal infection of the colon in a patient without concurrent human immunodeficiency infection: a case report and literature review

**DOI:** 10.1007/s10096-021-04268-5

**Published:** 2021-05-13

**Authors:** Alvaro Quincho-Lopez, Noah Kojima, John M. Nesemann, Rogger Verona-Rubio, Dina Carayhua-Perez

**Affiliations:** 1 San Fernando Medical School, Universidad Nacional Mayor de San Marcos, Lima, Peru; 2 Department of Medicine, David Geffen School of Medicine, University of California, Los Angeles, CA USA; 3 David Geffen School of Medicine, University of California, Los Angeles, CA USA; 4 Department of Pathology, Hospital Nacional Arzobispo Loayza, Lima, Peru; 5 Department of Pathology, Hospital Nacional Daniel Alcides Carrion, Lima, Peru

**Keywords:** Cryptococcal infection, Colon, Non-HIV

## Abstract

Cryptococcosis is a fungal infection that is rarely reported in patients without human immunodeficiency virus (HIV) infection, especially when the central nervous system (CNS) or pulmonary system is not involved. We report a case of isolated colonic cryptococcosis without disseminated disease in a 64-year-old immunocompetent woman without HIV infection who presented with chronic diarrhea and no episodes of fever or weight loss. The diagnosis was based on histopathology examination. Furthermore, we performed a literature review showing that few reports have been published so far and in the case of colonic cryptococcal infection, the prognosis is favorable among HIV-uninfected patients.

## Introduction

*Cryptococcus* spp. are the causative agents of cryptococcosis, a fungal infection that occurs worldwide. Cryptococcosis is one of the leading causes of death among immunosuppressed individuals, especially among those infected with human immunodeficiency virus (HIV) [1]. Although *Cryptococcus neoformans* causes more than 90% of cryptococcal infections, *Cryptococcus gattii* affects a greater proportion of immunocompetent individuals, and has a high prevalence in Latin America [1, 2].

*Cryptococcus* spp. can infect the central nervous system (CNS) and the pulmonary system; however, it can spread to any organ system, especially among cases of severe immunosuppression. Despite this ability to infect any organ system, colonic cryptococcosis that spares other digestive organs is rare, especially among immunocompetent persons [2].

Herein, we report a case of isolated colonic cryptococcosis without disseminated disease in an immunocompetent patient without HIV infection. Additionally, we performed a literature review of other cases of cryptococcosis that have involved the colon, either individually or as part of a disseminated disease, among non-HIV-infected patients.

## Case presentation

A 64-year-old woman with a history of high-grade medullary thyroid carcinoma and untreated asthma presented to the Arzobispo Loayza Hospital in Lima, Peru with a chief complaint of 5 months of intermittent chronic diarrhea. She also reported sporadic diffuse abdominal pain that occurred 1 month ago, rectal bleeding, and a painful ano-rectal mass, without fever or weight loss.

On admission, her heart rate was 89 beats/min, respiratory rate of 15 breaths/min, blood pressure of 115/80 mm Hg, temperature of 37.6°C, and her oxygen saturation was 98%. On physical examination, her abdomen was symmetric, soft, and non-tender without distention. Bowel sounds were present. No masses, hepatomegaly, or splenomegaly were noted. On the left side of her thyroid, a painless mass without lymphadenopathy was noted. Laboratory results were as follows: hemoglobin: 12.5 g/dL; WBC: 6160 cell/mm^3^ with 12% eosinophils (absolute eosinophil count, 739); platelets: 317,000/mm^3^; total proteins: 7.5 g/dL; albumin: 4.3 g/dL; INR: 0.9; basal glycemia: 97 mg/dL; glycosylated hemoglobin: 5.5%. ELISA HTLV-1 and ELISA HIV-1 were non-reactive. In addition to serologic testing, the HIV RNA assay was negative. Serum creatinine and liver function tests were normal. Chest radiography was negative for nodules, hilar lymphadenopathy, and pleural effusions.

**Fig. 1:**
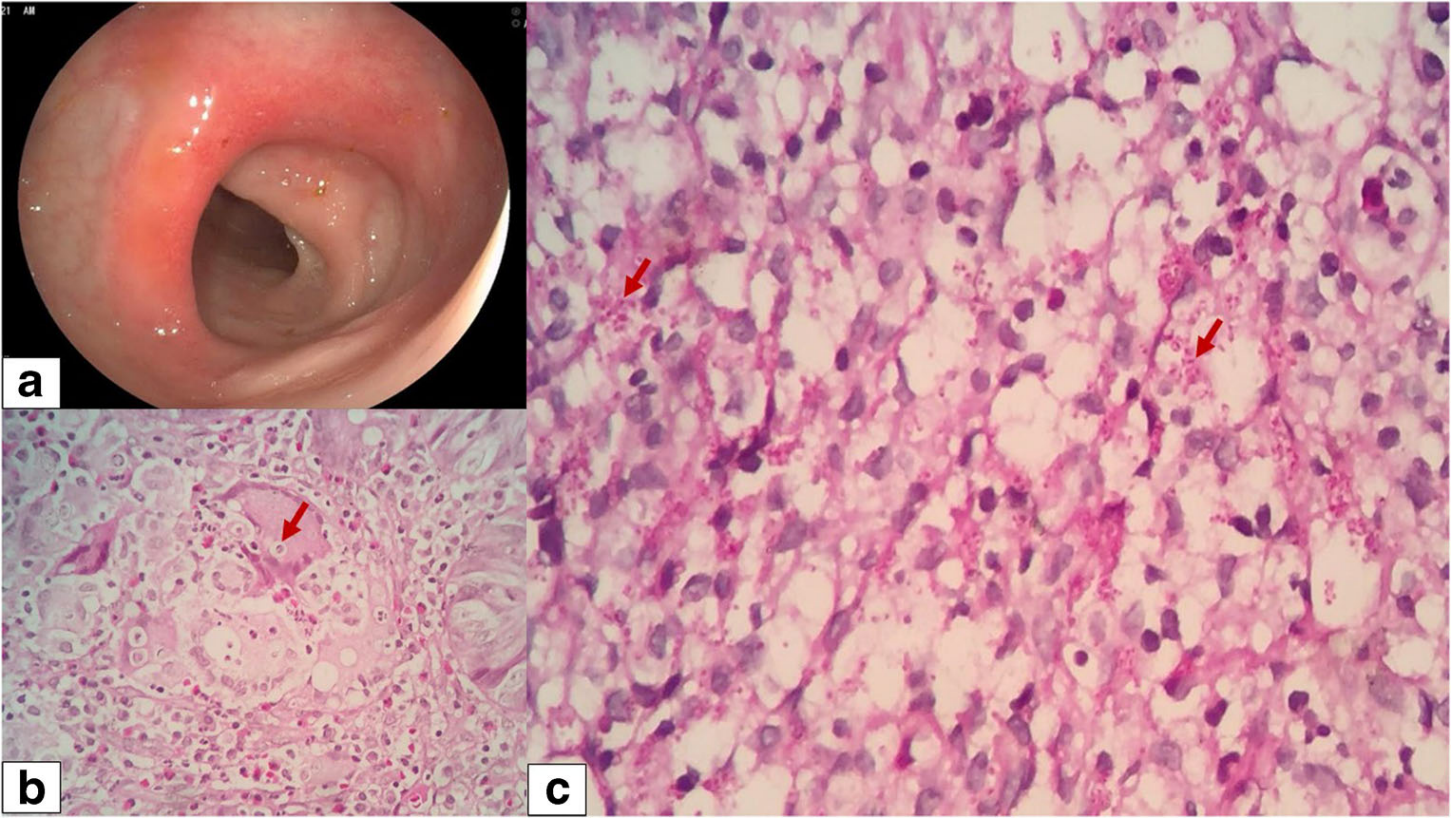
**a** Colonoscopy: Erythematous area is observed with the presence of small diffuse erosions circumscribed by normal mucosa in the central part of the haustra in the sigmoid colon. **b** Hematoxylin and eosin (H&E) stain shows a foreign body granuloma and multinucleated giant cells phagocytizing intracellular spherical structures that measure between 3 and 15 μm surrounded by a capsule of variable thickness that corresponds to *Cryptococcus* spp. (at magnification of ×400). **c** Periodic acid-Schiff (PAS) stain shows a conglomerate of histiocytes containing intracytoplasmic spherical structures corresponding to *Cryptococcus* spp. (at magnification of ×100)

A colonoscopy was performed and described segmental erosive sigmoiditis (Fig. 1a). In the sigmoid colon, 30 cm from the anal margin, a congestive and eroded mucosa was evident on the fold, with loss of the submucosal vascular pattern that compromises 60% of the circumference, not more than 2 cm in length. Grade III internal hemorrhoids were also observed.

Histopathological examination of the colonic tissue using hematoxylin and eosin (H&E) and periodic acid-Schiff (PAS) staining (Fig. 1b and 1c, respectively) revealed superficial mucosa with moderate acute and chronic inflammatory infiltrate, presence of granulomas, with multinucleated giant cells and PAS-positive thick-walled ovoid structures, consistent with *Cryptococcus* spp. Serum latex agglutination test for cryptococcal antigen (CrAg) was non-reactive. Further workup such as blood culture and cerebrospinal fluid (CSF) examination through lumbar puncture was negative for fungal or bacterial infection, being the patient diagnosed as an isolated colonic cryptococcosis case without disseminated disease. She started antifungal therapy with fluconazole 400 mg/day for 6 months with clinical improvement.

## Discussion

The diagnosis of cryptococcosis is based on the direct visualization of the fungus with an India ink stain, culture of biological samples such sputum or CSF, histopathologic staining of tissues, or serological tests that detect the presence of the cryptococcal polysaccharide capsular antigen (CrAg) [1, 2]; the latex agglutination test and lateral flow assay (LFA) can be used in both serum and CSF samples [3, 4]. Our patient serological assays were non-reactive, so we chose to obtain a biopsy for histologic diagnosis.

Few cases of HIV-negative colonic cryptococcal infection have been published so far; we found 12 case reports in our literature review (Table 1). In these cases, a majority of the patients were female (58.3%; 7/12) and 66.6% (8/12) had a comorbidity, the most common of which was Crohn’s disease (25%; 3/12), which is often treated with immunosuppression [11, 12, 15]. The other 4 patients presented without any comorbidities [6, 9, 10, 16]. Diarrhea was the most frequent presenting symptom, occurring in 50% of the patients [6, 11–13, 15, 16], followed by abdominal pain (41.6%; 5/12) [6, 11, 12, 15, 16], and fever (33.3%; 4/12) [8, 11, 12, 15]. These three symptoms occurred simultaneously in 25% (3/12) of the reported cases [11, 12, 15]. Only one patient was asymptomatic on presentation and was diagnosed incidentally [14]. The ascending colon was affected in 41.6% (5/12) of the cases [5, 7, 10, 14, 15]. The types of lesions observed during colonoscopy were ulcers [5, 11–15] and masses [6–8, 10, 15], in 50% (6/12) and 41.6% (5/12) of the patients, respectively. Disseminated disease (> 1 noncontiguous site) was found in four (33.3%; 4/12) patients: one in skin [8], one in lungs [15], and two in the CNS [5, 11]. Amphotericin B (AmB) plus fluconazole (FCZ) was the preferred therapy in 33.3% (4/12) of the cases [10, 13, 16], and all cases that received this treatment improved clinically; 16% (2/12) of patients received AmB plus flucytosine (5FC) [8, 11], with mixed results, as one patient died (8.3%; 1/12); the rest of the cases were managed with FCZ monotherapy (8.3%; 1/12) [9], or surgery plus AmB (16.6%; 2/12) [6, 7], all with favorable results. There were two (16.6%; 2/12) deaths, one from respiratory failure [5] and another from multiple organ failure [8].

**Table 1:** Colonic cryptococcal infection case reports in non-HIV patients

Reference	Sex/age (years)	Underlying conditions	Clinical presentation	Type of lesion	Colonic distribution	Another organ involvement	Treatment	Outcome
Zelman [5]	M/25	CML, chemotherapy	NR	Ulcer	Ascending and transverse colon	CNS and visceral infiltration	None	Died
Unat [6]	M/16	None	Abdominal pain, diarrhea, LGIB	Mass	Descending colon	No	Sx + AmB	Resolved
Hutto [7]	F/29	Job’s syndrome	Chronic perirectal abscess	Stricture, mass	Ascending colon, perirectal area	No	Sx + AmB	Resolved
Daly [8]	M/63	Cirrhosis, splenectomy, corticosteroids	Fever, chills, peritonitis, skin lesions	Mass	Transverse colon	Skin and omentum	AmB + 5FC	Died
Melato [9]	F/84	None	Rectal bleeding	Pedunculated polyp	Sigmoid colon	No	Polypectomy	Resolved
Song [10]	F/27	None	Melena	Mass	Ascending colon	No	AmB + FCZ	Resolved
Osawa [11]	M/53	Silicosis, Crohn’s disease (INX, prednisone, AZA)	Fever, abdominal pain, and diarrhea	Ulcer	Cecum	CNS	AmB + 5FC	Resolved
Sciaudone [12]	F/26	Crohn’s disease	Abdominal pain, fever, diarrhea, melena, weight loss	Ulcer, patchy lesions	Sigmoid colon, transverse colon, and cecum	NR	FCZ	Resolved
Cicora [13]	F/59	Hypertension, Chagas disease, and kidney transplant	Diarrhea	Ulcer	NS	NR	AmB + FCZ	Resolved
Túlio [14]	M/70	Madelung disease, hypertension, diabetes, adenocarcinoma (pancreas)	None	Ulcer, stricture	Ascending colon	No	NR	NR
Chavapradit [15]	F/58	Crohn’s disease (prednisolone, AZA, MZ)	Abdominal pain, fever, and diarrhea	Ulcer, mass	Ascending colon, ileocecal valve	Lungs	AmB + FCZ	Resolved
Medina Alvarez [16]	F/57	None	Abdominal pain, diarrhea, hematochezia	Nodular lesions	From rectum to descending colon	NR	AmB + FCZ	Resolved

Note. *AmB* amphotericin B; *AZA* azathioprine; *CML* chronic myeloid leukemia; *CNS* central nervous system; *FCZ* fluconazole; *F* feminine; *INX* infliximab; *L* lower gastrointestinal bleeding; *M* masculine; *MZ* mesalazine; *NR* not reported; *NS* not specified; *Sx* surgery; *5-FC* flucytosine

In a more general review of fungal infections in the colon, 77% of cryptococcosis occurred in immunosuppressed patients (either with HIV infection or on immunosuppressive therapy), and more than half developed disseminated disease [17]. Although many of the symptoms it produces are general, the most specific is perirectal abscess [7], *Cryptococcus* spp. being the only fungus that invades the perirectal area [17].

According to the 2010 Infectious Diseases Society of America (IDSA) clinical practice guidelines, for non-disseminated cryptococcal disease that does not involve the CNS or lungs, 400 mg of oral fluconazole per day for 6-12 months is recommended [18]. Although overall response to antifungal therapy is variable, all reports of patients without HIV infection or disseminated disease demonstrate favorable response to treatment [17]. This is consistent with our review of the literature, since only two patients [5, 8], both with disseminated disease, passed away. This suggests that regardless of the treatment received, the prognosis is generally favorable among patients without HIV infection.

Some limitations that we must emphasize are that the patient was not further screened for primary immunodeficiencies and only some of the most common secondary immunodeficiencies (i.e., diabetes, malnutrition) were ruled out.

Clinicians should be aware that cryptococcal infection can occur among immunocompetent patients without neurologic or systemic compromise. Here we described an unusual case of a patient presenting with chronic diarrhea. Histopathological diagnosis is essential, even more so if serological methods are non-reactive, as in our case. Among HIV-uninfected patients, the diagnosis is usually late, although in the case of colonic infection, the prognosis is favorable.

## Data Availability

Data sharing is not applicable to this article as no datasets were generated or analyzed during the current study.
